# Professor Desmarres, of Paris

**Published:** 1847-02

**Authors:** 


*Professor Desmarres, of Paris.*—From the warm and hearty expressions of gratitude manifested by all the American medical stutents who have had the advantage of this eminent man's teaching, it is apparent that he is a rising star in the great theatre of European surgery. Courtesy of manner towards strangers and pupils, in a medical teacher, will do more than gold in laying a foundation for distinction. This gentleman's kindness of manner, and devotedness to the best interests of Americans who visit the medical and surgical institutions of Paris, have secured for him in the United States a great body of friends, who will keep in perpetual remembrance his civilities, as well as his untiring efforts to place those who seek his assistance in the very focus of surgical knowledge. We are gratified to be able to make honorable mention of such uniform attentions to our countrymen.—*Boston Med. and Surg. Jour.*

